# Supplementary material for: Influence of Traditional vs Alternative Dietary Carbohydrates Sources on the Large Intestinal Microbiota in Post-Weaning Piglets
Source: Animals (Basel). 2019 Aug 1;9(8):516. doi: 10.3390/ani9080516 (PMC6719221; doi:10.3390/ani9080516)
Supplement: Supplementary file 1 [file animals-09-00516-s001.pdf]

Supplementary Materials:

Supplementary Table 1. Ingredient composition (g/100 g of diet) of the two experimental diets.

Ingredients	CTR	FFPs
Former Foodstuffs	-	30
Barley	22.8	22.1
Dextrose	5	4.5
Flaked decorticated barley	4	0
Corn	6.5	4
Flaked corn	6.5	1
Vegetable fibres	1	1
Wheat	12.3	10.1
Flaked wheat	6	1
Wheat bran	3	2.48
Vegetable oil	1.5	0.5
Soy oil	1.5	0.5
Fish meal (65% protein)	2.5	2.6
Plasma powder	3.5	3.8
Whey powder	11	4.5
Soy e.f. 50 [1]	3.5	3.5
Soycomil R [2]	5.5	4.55
L-lysine HCl	0.55	0.55
DL-methionine	0.23	0.23
L-threonine	0.25	0.25
L-tryptophan	0,08	0.08
Vitamin-mineral premix [3]	2.76	2.76
Total	100	100

CTR = standard diet; FFPs = former foodstuffs products diet; [1] Soy extraction flour 50%; [2] High quality soy protein concentrate; [3]Provided per 100 g of complete diet: 0.25 g Vitaminic premix, 0.4 g Benzoic acid, 0.5 g Hydrated dicalcium phosphate, 0.4 g Calcium carbonate, 0.15 Sodium chloride, 0.8 g Acidifying mixture, 0.06 g Copper sulphate, 0.2 g Sodium butyrate

Supplementary Table 2. Analysed composition (g/100 g or MJ/kg on DM) of the CTR and FFP diets.

Dietary treatments	CTR	FFPs
Dry matter	90,9	90,3
Ash	5,60	5,42
CP	20,9	20,6
EE	5,94	5,92
Crude fibre	4,20	2,80
NDF	13,1	9,56
ADF	3,50	2,86
Total dietary fibre	16.0	14.0
Insoluble fibre	14.0	11.6
Soluble dietary fibre	1.90	2.30
Starch	36,2	42,6
Glucose	6.00	6,17
Fructose	0,13	0,41
Sucrose	1,07	3,11
Lys *	1,55	1,53
Met + Cys *	0,93	0,90
Calculated metabolisable energy	16	16

CTR = standard diet; FFPs = former foodstuffs products diet in which 30% of FFPs partially replaced conventional cereal grains; CP = crude protein; EE= ether extracts; NDF = neutral detergent fibre; ADF = acid detergent fibre; Lys = lysine; Met = methionine; Cys = cysteine; * = calculated value.

Supplementary Table 3. Per sample abundance of sequences after the quality check of reads.

Sample ID	Counts/sample	Diet
158886F143366	24863	FFPs
158883F143363	36202	CTR
158889F143369	37764	CTR
150409F143359	39688	FFPs
158885F143365	41484	FFPs
150410F143360	42167	CTR
150412F143362	43806	FFPs
158884F143364	49435	CTR
150411F143361	54813	CTR
158887F143367	55801	FFPs
158888F143368	80702	CTR
158894F143374	24390	CTR
158899F143379	36652	CTR
158891F143371	38391	CTR
158900F143380	44260	CTR
158892F143372	48623	CTR
158895F143375	54295	CTR
158890F143370	54651	FFPs
158893F143373	57223	FFPs
158897F143377	62320	FFPs
158896F143376	70288	FFPs
158902F143382	23829	CTR
158910F143390	27917	CTR
158907F143387	43826	FFPs
158911F143391	48854	CTR
158905F143385	51813	CTR
158908F143388	55577	FFPs
158901F143381	56299	FFPs
158906F143386	58364	CTR
158904F143384	62769	FFPs
158909F143389	63261	FFPs
158903F143383	64890	CTR

CTR= standard diet; FFPs = former foodstuffs products diet in which 30% of FFPs partially replaced conventional cereal grains.

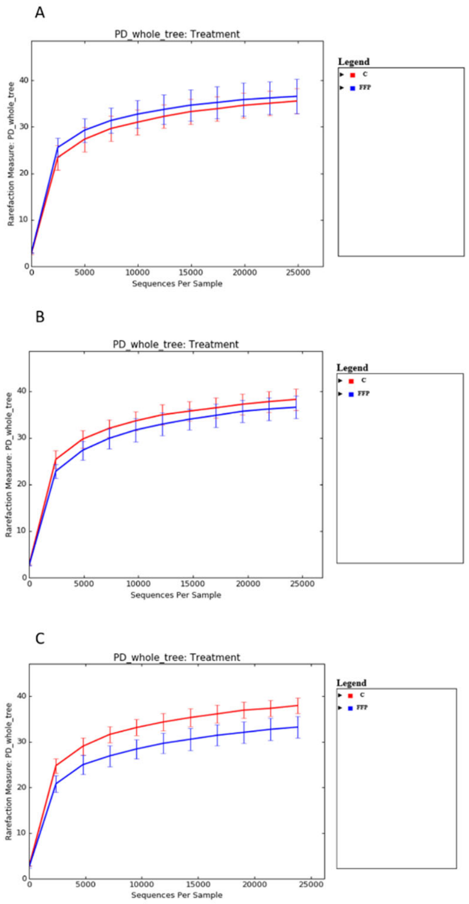

Supplementary figure S1. Rarefaction plots per group in different time points. The lines for categories that extend all the way to the right end of the x-axis means that all the samples in that category have the same number of sequences. A) Rarefaction plot of the two categories at day 0 (D 0); B) at day 8 (D 8); C) at day 16 (D 16).
